# Streamlining Homogeneous Glycoprotein Production for Biophysical and Structural Applications by Targeted Cell Line Development

**DOI:** 10.1371/journal.pone.0027829

**Published:** 2011-12-09

**Authors:** Sonja Wilke, Lothar Groebe, Vitali Maffenbeier, Volker Jäger, Manfred Gossen, Jörn Josewski, Agathe Duda, Lilia Polle, Raymond J. Owens, Dagmar Wirth, Dirk W. Heinz, Joop van den Heuvel, Konrad Büssow

**Affiliations:** 1 Department of Molecular Structural Biology, Helmholtz Centre for Infection Research, Braunschweig, Germany; 2 Department of Experimental Immunology, Helmholtz Centre for Infection Research, Braunschweig, Germany; 3 Max Delbrück Center for Molecular Medicine (MDC), Berlin, Germany; 4 Berlin-Brandenburg Centre for Regenerative Therapies (BCRT), Berlin, Germany; 5 Division of Structural Biology, Henry Wellcome Building for Genomic Medicine, University of Oxford, Oxford, United Kingdom; 6 Oxford Protein Production Facility UK, The Research Complex at Harwell, Rutherford Appleton Laboratory Harwell Science and Innovation Campus, Oxfordshire, United Kingdom; 7 Department of Gene Regulation and Differentiation, Helmholtz Centre for Infection Research, Braunschweig, Germany; Bangor University, United Kingdom

## Abstract

Studying the biophysical characteristics of glycosylated proteins and solving their three-dimensional structures requires homogeneous recombinant protein of high quality.We introduce here a new approach to produce glycoproteins in homogenous form with the well-established, glycosylation mutant CHO Lec3.2.8.1 cells. Using preparative cell sorting, stable, high-expressing GFP ‘master’ cell lines were generated that can be converted fast and reliably by targeted integration via Flp recombinase-mediated cassette exchange (RMCE) to produce any glycoprotein. Small-scale transient transfection of HEK293 cells was used to identify genetically engineered constructs suitable for constructing stable cell lines. Stable cell lines expressing 10 different proteins were established. The system was validated by expression, purification, deglycosylation and crystallization of the heavily glycosylated luminal domains of lysosome-associated membrane proteins (LAMP).

## Introduction

Structural and biophysical studies of glycosylated proteins require recombinant protein samples of high quality and homogeneity. Production of glycoproteins relies mostly on eukaryotic protein expression systems [1]. Protein-linked glycan chains are essential for protein folding and secretion, but they cause sample heterogeneity, which complicates protein crystallization and biophysical measurements, e.g. determination of molecular mass and oligomerization status [2]. Inhibitors and mutations of N-acetylglucosaminyl-transferase I (GnTI) prevent the processing of N-linked glycans beyond the high-mannose type, leading to smaller and more homogeneous modifications [3], [4]. GnTI-negative HEK293 and CHO Lec [3] cell lines have enabled the crystallization of a number of glycoproteins [5], [6], [7]. High-mannose type glycans can be truncated efficiently to a single N-acetylglucosamine by endoglycosidase H, which usually does not affect protein stability, but often improves crystal growth [4], [8].

**Figure 1:**
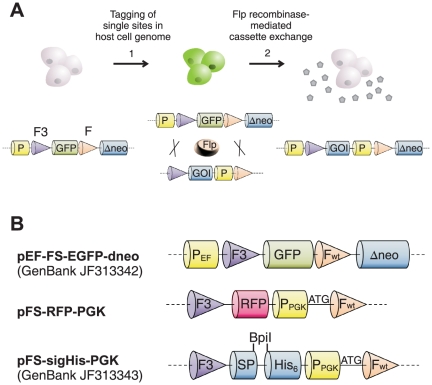
Strategy and vector maps. (A) Strategy for establishing production cell lines by RMCE. The tagging vector contains an EF promoter (P) controlling the expression of a GFP gene flanked by a set of heterospecific FRT sites, the synthetic variant F3 and the wild-type F. A silent, ATG-deficient neomycin resistance (Δneo) gene allows selection of targeted cells. (1) CHO Lec3.2.8.1 host cells are transfected with the tagging vector. GFP-tagged cells are isolated by two rounds of FACS. (2) Cassette exchange is initiated by co-transfecting a tagged cell line with a Flp expression vector and a targeting vector bearing the gene of interest (GOI) and a PGK promoter (P) destined to complement the Δneo gene. These genetic elements are flanked by FRT sites compatible to the tagging vector. Thus, the transiently expressed Flp recombinase exchanges the tagging gene cassette. New production cell clones with chromosomally integrated GOI are selected by G418. (B) The tagging and targeting vectors used in this study. SP = signal peptide.

Stable cell lines with good performance have integrated the recombinant transgene at a genetically stable hot spot of transcription. Preparative fluorescence-activated cell sorting (FACS) is very efficient for isolating such cell lines [9], [10], [11], [12] and was applied by us previously to glycosylation mutant CHO cells for crystallization of glycoproteins [8]. However, preparative sorting of CHO cells growing in suspension can be challenging, especially if cell lines for several target proteins have to be established in parallel. Therefore, in this study, we combined cell lines carrying a fluorescent marker at a hot spot of transcription with targeted gene integration, thus allowing to derive production cell lines for arbitrary proteins from the same fluorescent master cell line in a single step (Fig. 1A).

Genome engineering by recombinase-mediated cassette exchange (RMCE) allows targeted integration of transgenes precisely into defined expression hot spots of the host cell genome [13], [14]. RMCE with the recombinase Flp requires a master cell line ‘tagged’ at such a hot spot by a reporter gene cassette flanked by Flp recognition target (FRT) sites. The flanking FRT sites, the wild type and a synthetic variant, cannot recombine with each other. RMCE is achieved by co-transfecting the tagged master cell line with a targeting vector containing the gene of interest, flanked by the same pair of FRT sites, and a Flp expression vector (Fig. 1A). A double-reciprocal crossover of the FRT sites leads to an exchange of the reporter with the gene of interest in the host cell genome. RMCE is thus practically irreversible, in contrast to recombination systems that use only a single recombination site [15], [16]. For antibiotic selection of recombinant cell lines, RMCE has been combined with a selection trap, consisting of a truncated antibiotic resistance marker that becomes complemented by the recombination event [17]. 

In the present study, GFP-positive master cell lines were established by preparative cell sorting that allows integrating genes of interest by RMCE with selection trap (Fig. 1A). Stable production cell lines for 10 different proteins were established, including members of the heavily glycosylated lysosome-associated membrane protein (LAMP) family. The system was validated by expression, purification, deglycoslation and crystallization of different LAMP luminal domains.

## Results

### Glycosylation mutant RMCE master cell lines

Stable RMCE master cell lines were derived from the CHO Lec3.2.8.1 glycosylation mutant [3] by transfection with the vector pEFFS-EGFP-dneo (Fig. 1B) that contains a GFP reporter gene under control of the human elongation factor 1α (EF) promoter (Fig. 1). The GFP gene is flanked by the wild type FRT site (F) and the synthetic variant F3. A selection trap, an ATG-deleted, promoterless neomycin phosphotransferase (Δneo) gene is located downstream of GFP. When GFP is exchanged by Flp-mediated recombination against a cassette including a promoter and start codon, Δneo becomes complemented and the cell becomes resistant against the antibiotic G418, thus allowing for selection of recombinant cells. 

**Figure 2:**
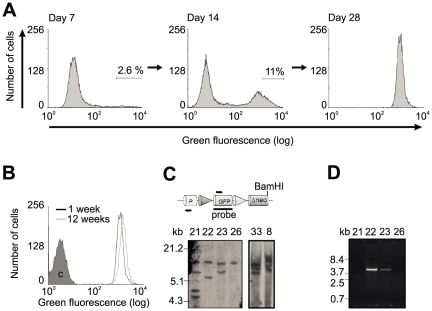
Generation of master cell lines. (A) Selection of CHO Lec3.2.8.1 cells upon transfection with the GFP tagging vector pEF-FS-EGFP-dneo. One week post transfection, the top 2.6% fluorescent cells were isolated. Of these cells, the top 11% fluorescent cells were isolated as single cells one week later. The fluorescence profile of a representative cell clone is shown. (B) Fluorescence profile of a representative tagged cell clone in comparison to parental CHO Lec3.2.8.1 cells (marked with ‘C’). The GFP fluorescence of the tagged cells was observed over 12 weeks without measuring a reduction in fluorescence strength. (C) Southern blot analysis of integrated tagging vector copy numbers in six potential master cell clones. Genomic DNA was digested by BamHI, blotted and probed for GFP. Multiple bands indicate integration at multiple chromosomal sites. (D) PCR test for concatemers in four potential master cell clones. Primers are marked by horizontal arrows in panel C. PCR products were obtained only in the presence of tandem repeats. Cones = FRT sites (dark = F3, light = wild type).

CHO Lec3.2.8.1 cell clones with stable genome integration of pEFFS-EGFP-dneo were isolated by two rounds of preparative FACS. The 2.6% most highly fluorescent cells were isolated one week post transfection (Fig. 2A). One week later, 11% of the isolated cells had retained strong fluorescence and were again isolated by FACS. Transfection and sorting was repeated once and 1.1% and 5% of the cells were isolated in the first and second round of FACS, respectively. 30 cell clones obtained from each transfection were cultured for 12 weeks and 15 cell clones with stable GFP expression and favourable growth characteristics were finally isolated (Fig. 2A,B). 

Multiple integrated transgene copies can lead to irreproducible results in RMCE. Therefore, the number of genetic loci bearing the pEFFS-EGFP-dneo transgene was determined by Southern blot (Fig. 2C) and the presence of tandem repeat concatemers was tested by PCR (Fig. 2D). Two out of seven tested cell lines were found to contain a single copy of the transgene (SWI3a-26, SWI3b-5, Table 1).

**Table 1:** Recombination and production properties of different master cell clones and RFP subclones.

Master cell clones	SWI3a-22	SWI3a-23	SWI3a-26	SWI3a-33	SWI3b-5	SWI3b-18	SWI3b-25
Transgene copy number	2	2	1	3	1	3	2
Concatemers	yes	yes	no	no	no	no	no
GFP concentration [mg l^−1^]	19	14	12	11	20	30	21
Proportion of subclones retaining GFP after RMCE	0/6	2/5	0/3	6/6	0/2	0/2	0/3

### Exchange of GFP against RFP

**Figure 3:**
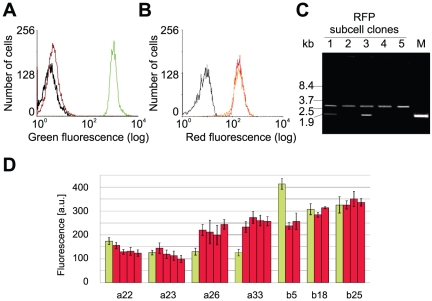
Exchange of GFP with RFP by RMCE. (A) Green fluorescence of a GFP-tagged master cell clone (green), a GFP-negative RFP subclone (red) and CHO Lec3.2.8.1 cells (grey). (B) Red fluorescence of an RFP subcell clone upon one-week (orange) and 5-week (red) cultivation, compared to the corresponding master cells (grey). (C) Verification of RMCE by PCR amplification of the FRT-flanked gene cassette (GFP 1.9 kb, RFP 2.7 kb) from chromosomal DNA of cell lines derived from a master cell line with multi-copy transgenes. Lanes 1–5 and M represent 5 representative RFP positive subcell clones and the master cell line. Cell lines with incomplete exchange of integrated GFP gene copies were detected in lanes 1 and 3. (D) Expression strength of 7 GFP master cell clones and 2–4 corresponding RFP targeted subcell clones. Intracellular GFP (green) and RFP (red) fluorescence was measured by flow cytometry. GFP fluorescence was scaled down by a factor of 10.

GFP was exchanged in seven master cell lines against a red fluorescent protein (RFP) gene by RMCE, performed by co-transfecting the master cell lines with an expression plasmid for the highly active Flp variant FLPo [18], [19] and the exchange vector pFS-RFP-PGK (Fig. 1B) . The RFP gene present in pFS-RFP-PGK lacks a promoter and was inactive until it was positioned by recombination adjacent to the EF promoter present in the master cells (Fig. 1). Upon recombination, a PGK promoter and a start codon located on the exchange vector activated the Δneo gene at the tagged locus. Resistant colonies were obtained at a frequency of about 3×10^−5^ (40–50 colonies from 1.5×10^6^ transfected cells). Several resistant cell clones were isolated per master cell line and red fluorescence was detected by FACS, which was stable over at least five weeks (Fig. 3B). The RFP cassette was detected in all subclones by PCR (Fig. 3C). Of the five master cell lines carrying multiple copies of the GFP transgene, two gave rise to subclones that had retained GFP copies in their genome, indicating incomplete RMCE (Fig. 3C, Table 1). These GFP gene copies were obviously inactive as green fluorescence was not detectable. GFP was absent in RFP clones derived from the other three multi-copy master cell lines, indicating that all of the two or three GFP copies present in these cells were exchanged in the RMCE reactions (Table 1). Random integration of the RFP exchange plasmid or the Flp expression vector was not detected by Southern blot and PCR analysis of 5 and 12 subclones, respectively (data not shown). 

GFP and RFP expression of seven master cell clones and their corresponding subcell clones was quantified (Fig. 3D). GFP from four-day master cell cultures varied between 11 and 30 mg/l according to fluorescence spectroscopy of cell extracts (Table 1). GFP concentrations corresponded well to flow cytometry intensities. Subclones of the same origin produced similar amounts of RFP, as expected for isogenic cells (Fig. 3D). The two master cell lines containing transgene concatemers, SWI3a-22 and SWI3a-23, gave rise to cell lines with notably low RFP expression, which can be explained by the reduction of the concatemers to single copies during the recombination reaction. RMCE with the remaining five master cell lines, although these produced different amounts of GFP, resulted in subcell clones expressing similar amounts of RFP. This suggests that factors other than transcriptional activity limited the maximal RFP concentration in this cell type.

### Protein production cell lines established by RMCE

**Figure 4:**
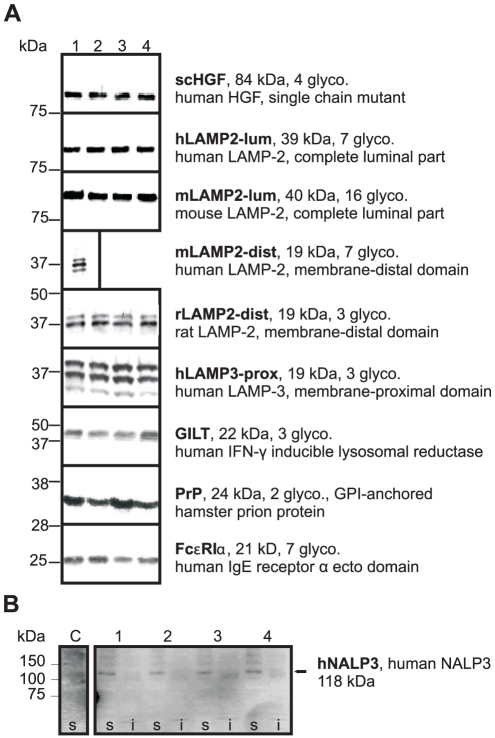
Recombinant protein expression by production cell lines derived by cassette exchange. (A) Immunoblots of supernatants containing secreted recombinant glycoproteins. Molecular weights were calculated without signal peptides, glycosylations and GPI-anchors. The number of glycosylation sites is indicated (glyco.). Each lane represents a different subcell clone. Four subcell clones are shown for each protein with the exception of mLAMP2-dist, for which only one subcell clone was isolated. (B) Western blot of cell lysates of four NALP3 targeted subcell clones. Small amounts of recombinant human NALP3 were detected in all four soluble lysate fractions of the targeted cells, but not in the SWI3a-26 master cells (C). i = insoluble, s = soluble.

Most production cell lines were derived from master cell line SWI3a-26, which was genetically stable, carried a single-copy transgene of high transcriptional activity, performed well in RMCE and gave rise to highly fluorescent RFP subclones. Cell lines for 10 different target proteins were established to produce protein for crystallization, including members of the family of lysosome associated membrane proteins (LAMPs). The LAMPs' large N-terminal portion is highly glycosylated and resides in the lysosomal lumen. It is composed of two similar domains, which are connected by a proline-rich, flexible hinge. A set of 33 LAMP domains of different species was cloned into the mammalian expression vector pEFFS-sigHA with a C-terminal hemagglutinin (HA) tag for immunodetection (Table S1). HEK293 and CHO Lec3.2.8.1 were transiently transfected and secreted proteins were detected by immunoblotting (data not shown). Five sequences were selected for construction of stable cell lines: the full luminal region of human and mouse LAMP-2 (termed ‘hLAMP2-lum’ and ‘mLAMP2-lum’), the membrane-proximal domain of human LAMP-3, also known as DC-LAMP (‘hLAMP3-prox’) and the membrane-distal domains of mouse and rat LAMP-2 (‘mLAMP2-dist’, ‘rLAMP2-dist’). In addition, cell lines were generated for an engineered single chain (sc) variant of hepatocyte growth factor (HGF, also known as scatter factor), hamster prion protein (PrP), high affinity IgE receptor subunit α (FcεRIα), γ-interferon inducible lysosomal reductase (GILT) and NALP3 (Fig. 4). 

RMCE was performed as described for RFP. Depending on the construct, the optimal mass ratio of co-transfected targeting vector and Flp-expression vector was 1∶4, 2∶3 and 1∶1, corresponding to a molar ratio of about 1∶2. Four subclones were typically expanded for each construct, and RMCE was confirmed by PCR. Recombinant protein production by correctly targeted cell clones was analyzed by Western blot (Fig. 4). Sublones derived from the same RMCE reaction expressed their transgene at a similar level, as expected for isogenic cells. With the exception of NALP3 cell clones, recombinant protein yield appeared adequate for producing at least10 mg of purified protein in a 22 litre bioreactor run. This amount allows for extensive crystallization screening and optimization. NALP3 is an intracellular protein that was solubly produced and detected in small amounts by an anti-His antibody (Fig. 4B) and by an anti-NALP3 antibody (data not shown) as well.

An scHGF cell clone with high productivity and acceptable growth was chosen for further analysis. The clone produced 1.4±0.4 pg per cell per day (pcd) in 40 ml spinner flasks, similar to a previously described scHGF cell clone derived from a Flp-mediated reporter gene excision system [8] (SWI4_25a; 1.0±0.2 pcd) and considerably more than a conventionally established cell clone for wild-type HGF [20] (EGT92/A20; 0.5±0.1 pcd).

### Protein production and crystallization

**Figure 5:**
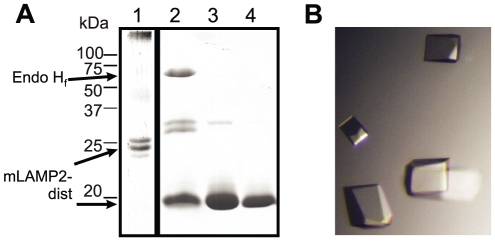
Protein purification and crystallization of LAMP domains. (A) Purified mLAMP2-dist with intact glycosylation (lane 1), deglycosylated mLAMP2-dist (lane 2) and deglycosylated mLAMP2-dist purified by gelfiltration (10 µg in lane 3 and 5 µg in lane 4) were analysed by SDS-PAGE and Coomassie staining. Additional mLAMP2-dist bands in lane 2 might have been caused by incomplete reduction of disulphide bonds. (B) Crystals of mLAMP2-dist.

Batches of up to 50 litre conditioned medium were produced by perfusion bioprocessing to obtain sufficient amounts of protein for crystallization. An scHGF production cell line derived by RMCE (SWI3a-26a) secreted 32 mg of the growth factor into 22.5 litre conditioned medium produced in perfusion mode. hLAMP3-prox and mLAMP2-dist were produced in the same way. From 22.5 litre culture supernatant, 27 mg hLAMP3-prox or 15 mg mLAMP2-dist were obtained upon diafiltration, affinity chromatography and gel filtration (Fig. 5A). mLAMP2-dist was deglycosylated with endoglycosidase H, subjected to sparse matrix crystallization screening and crystals were obtained (Fig. 5B). However, X-ray diffraction of the crystals tested so far was not sufficient to proceed with crystallographic analysis (Fig. 5A). Highly diffracting crystals of hLAMP3-prox had been described previously [8] and were also obtained from the protein produced by the RMCE cell line under the same conditions. Deglycosylation was required for crystallization, as no crystals could be grown of protein with intact glycosylation.

## Discussion

In this study, we describe glycosylation mutant master cell lines that reduce time and effort for high-quality stable cell line development. The combination of preparative cell sorting and RMCE was successful and is applicable to other mammalian cell line types as well. RMCE was applied for the first time to establish glycosylation mutant cell lines for protein production for X-ray crystallography. 

Master cell lines with single-copy transgenes were established that performed reliably in RMCE with RFP targeting vector. A promoter trap and a neomycin selection trap assured that the GFP reporter gene was absent in all the resulting cell clones (Fig. 1). A targeting frequency of about 3×10^−5^ was sufficient for robust generation of new production cell lines. Isolating large numbers of clones was not necessary since all targeted cell clones were isogenic and produced recombinant proteins at the same level.

**Figure 6:**
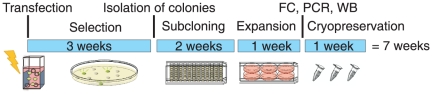
Timeline for RFP cell line development by RMCE. Following transfection, selection and subcloning, cells were analyzed by flow cytometry (FC) and PCR analysis to confirm the cassette exchange. Recombinant protein production was controlled with Western blots (WB) before cryopreservation of the cell clones. It took 7 weeks from transfection of the master cells to cryopreservation of the first aliquot of RFP cell clones.

Genetically stable, highly productive cell lines were reliably obtained that produced glycoproteins with limited glycosylation, amenable to enzymatic deglycosylation. Fig. 6 shows the time scale of establishing recombinant RFP cell lines by RMCE, which took 7 weeks from the day of transfection to cryopreservation of clonal production cell lines. Protein production with stable cell lines was scaled up reproducibly to large volumes according to the required amounts of protein. LAMP domains that could be produced well by stable cell lines were identified successfully by small scale, high-throughput transient transfections. 

RMCE with three master cell lines with multiple integrated transgenes resulted in complete exchange of all reporter gene copies to RFP. Obviously, simultaneous exchange at distinct loci had taken place in these cells. Simultaneous exchange at different loci has also been described recently in the context of multiplexing of RMCE with a novel set of synthetic FRT variants [21]. 

Previously, we established scHGF and hLAMP3-prox production cell lines by preparative cell sorting followed by Flp-mediated excision of the reporter [8]. scHGF cell lines established by RMCE had a similar, slightly higher specific productivity as these previously reported cells. Productivity was also similar in the case of the LAMP-3 domain. In comparison to the reporter gene excision approach, RMCE was considerably faster and more predictable. Fewer clones had to be analyzed and preparative cell sorting was not required. 

In conclusion, the system presented here simplifies generation of producer cell lines for homogenous glycoproteins. We demonstrate a strategy that combines high-throughput screening of genetic constructs by transient transfection with rapid establishment of stable cell lines. Large-scale protein production allowed purification and deglycosylation of milligram amounts of protein. High protein quality and homogeneity was proven by successful crystallization.

## Materials and Methods

### Plasmid construction

Coding sequences of 33 LAMP domains were cloned by PCR into the vector pEFFS-sigHA (GenBank HQ333206) between the vector encoded signal peptide and HA-tag sequences using the two Esp3I restriction sites of the vector. PCR products were either cloned with the In-Fusion system or by designing PCR primers with tails including Esp3I, Eco31I or BpiI sites that lead to sticky ends compatible to the vector upon digestion (Tables S1, S2).

The tagging vector pEF-FS-EGFP-dneo (GenBank JF313342) is based on pEF-FS-EGFP and contains GFP under control of the human EF1α promoter. The GFP gene is flanked by one synthetic (F3) and one wild type FRT site. An ATG-deleted neomycin phosphotransferase (Δneo) gene [22] was PCR amplified with primers 5′-TAGATGCATG CTCGAGCGAC TCTAGAGGAT CCCCCGA-3′ and 5′-AGGAACTTCG GAATTCAGTG GATTGCACGC AGGTTC-3′ and cloned between the EcoRI and XhoI sites of pEF-FS-EGFP with the In-Fusion cloning system (Clontech, Saint-Germain-en-Laye, France). For the construction of targeting vectors, the EF1α promoter was deleted by cutting pEF-FS-EGFP with BglII and HindIII, blunting the overhangs and ligating the free ends. Sequences encoding a signal peptide and a His-tag were cloned between the FRT sites, replacing GFP, resulting in pFS-sigHis. To obtain pFS-sigHis-PGK (GenBank JF313343), a PGK promoter and an ATG start codon [9], [23] were inserted upstream of the wild type FRT site to complement the inactive Δneo gene after targeting. pFS-RFP-PGK and the NALP3 exchange vector contain the open reading frames of dsRed RFP (GenBank ABB83400) or human NALP3 between the NcoI and MluI sites of pFS-sigHis-PGK. The following genes were cloned between the BpiI sites of pFS-sigHis-PGK for RMCE: human scHGF, human GILT (Swiss-Prot P13284), hLAMP3-prox (GenBank AAH32940), hLAMP2-lum, mLAMP2-lum, rLAMP2-prox, hamster PrP, the ectodomain of human high affinity IgE receptor α chain (FcεRIα) or human NALP3. The transgenes include a C-terminal His_6_-tag and a mouse immunoglobulin signal peptide (Swissprot P01750).

### Cell culture and transfection

A HEK293-6E cell line constitutively expressing the EBNA1 protein of EBV was obtained from the Canadian National Research Council, Montreal, Canada and was cultivated and transfected as described [21]. The glycosylation mutant CHO Lec3.2.8.1 cell line [3] was kindly provided by Dr. Pamela Stanley, Albert Einstein College of Medicine, New York. The cell line was adapted to growth in suspension in ProCho5 medium (Lonza, Cologne, Germany) supplemented with 11 mg/ml phenol red in spinner flasks at 37°C, 5% CO_2_ and 80 rpm or in multi-well plates at 37°C, 5% CO_2_ and 150 rpm on an Incutec K15-500 linear shaker (Barsbüttel, Germany). Cell numbers and viability were assayed by trypan blue dye exclusion method.

Plasmid DNA for transfection was purified with the EndoFree Plasmid Maxi Kit (Qiagen, Hilden, Germany). The tagging vector pEF-FS-EGFP-dneo was linearized with SalI followed by purification with the Plasmid Extract II Kit (Macherey-Nagel, Düren, Germany). 1.5×10^6^ cells in 2 ml medium were transfected with 1–5 µg linearized plasmid DNA by using program U-24 of the Amaxa nucleofection device according to manufacturer's guidelines (Lonza, Cologne, Germany; Nucleofector™ Kit V). 24 h post transfection the medium was exchanged and the cells were seeded into 6-well plates and cultivated at 37°C in a humidified atmosphere with 5% CO_2_ at 150 rpm. In the following days, the transfected cell cultures were expanded before entering the stationary phase.

### Flow cytometry and preparative FACS

CHO Lec3.2.8.1 cells were transported and sorted at room temperature. GFP expression was analyzed with a Guava EasyCyte Mini System (Guava Technologies, Hayward, CA, USA). Cells were stained with 50 µg/ml propidium iodide to exclude dead cells from the analysis. Preparative FACS was performed on a MoFlo high-speed cell sorter (Beckman Coulter, Krefeld, Germany). The sorter was equipped with an argon-ion laser tuned to 488 nm with 100 mW of power and an automated cell deposition unit for sorting into 96-well plates. GFP fluorescence was detected through a 530/40-nm bandpass filter. Data analysis was performed using CytoSoft 4.2 and WinMDI 2.9 software.

### RMCE

The cassette exchange in tagged CHO Lec3.2.8.1 cells was performed by Amaxa nucleofection (Lonza, Cologne, Germany; Nucleofector Kit V). Production cells lines were derived from SWI3a-26, except for LAMP2-lum and hLAMP3-prox cell lines, which were derived from SWI3a-33. The cells were co-transfected with 1 µg, 2 µg or 2.5 µg of the targeting vector and 4 µg, 3 µg or 2.5 µg of the optimized FLPo [18] expression vector Flpo-puro [23], respectively. The FLPo expression vector pPGKFLPobpA (Addgene plasmid 13793) was also used successfully. 24 h post transfection, the cells were seeded on a 100 mm culture dish in 10 ml CD-Hybridoma medium and cultivated at 37°C in a humidified atmosphere with 8% CO_2_. To select for the targeted subcell clones, 2 mg/ml G418 was added 4–5 days post transfection. Medium was replaced every three to four days until the subcell clones were picked in 96 well plates after two to three weeks. GFP negative cell colonies were expanded and recloned by serial dilution, if necessary. Clonal cell lines were adapted to suspension cultures in serum free ProCho5 medium (Lonza, Cologne, Germany) by adding 10 U/ml heparin (Sigma, Steinheim, Germany) during the first 2 passages.

### Southern blotting

Genomic DNA was analyzed by Southern blotting to identify the copy number of the integrated transgene. 10 µg chromosomal CHO DNA was digested with appropriate restriction enzymes and separated in 0.8% agarose gels together with a digoxigenin-labelled DNA molecular weight marker II (Roche, Mannheim, Germany) and transferred to Hybond N^+^ membranes (Amersham, Munich, Germany) o.n. by capillary transfer with 20× SSC buffer. After washing the membrane in 2× SSC buffer, the transferred DNA was fixed to the membrane by UV-crosslinking. Hybridisation and immunological detection was performed with DIG High Prime DNA Labeling and Detection Starter Kit II (Roche, Mannheim, Germany). A 755 bp GFP fragment, a 457 bp KpnI/PacI fragment from Flpo-puro [21] and a 667 bp NgoMIV/SacI of pFS-scHGF-PGK were used as hybridization probes for GFP, FLPo and PGK promoter sequences, respectively.

### PCR-based analysis of genomic DNA

Genomic DNA was isolated with an AquaGenomic™ kit (MoBiTec, Göttingen, Germany). For detection of integrated concatemers, PCRs were performed with primers specific for the tagging vector pEF-FS-EGFP-dneo (5′-GCAGCCAGGG GCGTGGAAGT AATTCAAGG-3′, 5′-TCACATGGTC CTGCTGGAGT TCGTGACCG-3′) and pointing towards the ends of the linearized vector. Thus, only chromosome-integrated vector concatemers gave rise to a PCR product with these primers. Following RMCE, exchange of the GFP marker was confirmed by PCR amplification of the FRT-flanked gene cassette with primers 5′-TCGGGAGATC TCGACCGAGC TTTGCAAA-3′ and 5′-TGCTCGAGCG GCCGCTCTAG AACTAGTGGA-3′. All PCRs were performed with 50 ng chromosomal DNA and the Expand High Fidelity PCR System (Roche, Mannheim, Germany).

### Fluorescence spectrometry

GFP concentration in RMCE master cell extracts was quantified by fluorescence spectroscopy with an Infinite M1000 fluorescence microplate reader (Tecan, Männedorf, Switzerland). 1.5×10^5^ cells/ml were seeded in 3 ml in 6-well plates or in 40 ml in spinner flasks. Fluorescence from the cell extracts was measured (excitation 470 nm, emission 510 nm) in non-fluorescent microtiter plates (Nunc, Langenselbold, Germany) using several dilutions of the samples, and was converted to 0.1–1 µg/ml standard curve of recombinant GFP standard (BioVision, Mountain View, CA, USA).

### ELISA

HGF product titres were quantified with the human HGF DuoSet ELISA Development System (R&D Systems, Abingdon, UK) and productivity per cell was calculated as described [8].

### Western blotting

Intracellular proteins were extracted by the Cytobuster™ protein extraction reagent (Novagen, Darmstadt, Germany). Cells expressing PrP conjugated to a GPI-anchor were solubilised in 10% (w/v) PBS supplemented with a protease-inhibitor cocktail (Roche, Mannheim, Germany) and 5 mM MgCl_2_ using a Dounce homogenisator. For Western Blot analysis, PrP lysates were treated with 400 kU/ml DNaseI (Roche, Mannheim, Germany).

Stable cell culture supernatants and PrP cell lysates were analyzed by 12% SDS-PAGE and Western blotting with a goat anti-HGF antibody (dilution 1∶1,000, R&D Systems, Abingdon, UK) for scHGF, a mouse anti-PrP antibody (dilution 1∶5,000, Prionics, Schlieren, Switzerland) for PrP and an anti-His antibody (dilution 1∶1,000, Novagen, Darmstadt, Germany) for all other proteins. The anti-HA antibody 12CA5 (1∶5,000, Roche) was used for transiently transfected cell supernatants. Detection was done with the respective secondary antibodies conjugated to an alkaline phosphatase (1∶2,000, Promega, Mannheim, Germany) or a horseradish peroxidase (1∶2,000, Dianova, Hamburg, Germany), before development with BCIP-NBT solution (Applichem, Darmstadt, Germany) or Super Signal West Pico chemiluminescent substrate (Thermo Scientific, Bonn, Germany), respectively.

### Purification and deglycosylation of LAMP domains

Production cell lines were cultivated in stirred tank bioreactors in perfusion mode and hLAMP3-prox and mLAMP2-dist were purified by nickel ion affinity chromatography and gelfiltration, as described previously [8]. mLAMP2-dist gelfiltration fractions in 10 mM HEPES-NaOH, pH 7.4, 150 mM NaCl were diluted to 20 mM NaCl with 20 mM Tris-HCl, pH 7.4 and loaded on a Mono Q anion exchange column. mLAMP2-dist was collected in the flow through and concentrated to 1.5 mg/ml with a 10 K Vivaspin concentrator (Sartorius Stedim Biotech, Göttingen, Germany). Domains were deglycosylated with a fusion of endoglycosydase H and maltose binding protein (Endo H_f_, New England Biolabs Inc., Beverly, MA, USA) and gelfiltrated again, as described [8]. mLAMP2-dist was deglycosylated at 1 mg/ml in 100 mM Na-acetate, pH 5.2, with 50 units Endo H_f_ per µg mLAMP2-dist.

### Crystallization

hLAMP3-prox was crystallized as described [8]. Crystallization screens for mLAMP2-dist were set up manually in 24-well format by preparing hanging drops of 200 nl protein at 25 mg/ml in (10 mM HEPES, pH 7.4, 150 mM NaCl) with an equal volume of reservoir buffer of the JCSG+ screen (Qiagen). Single crystals were obtained in 2 µl droplets composed of 1 µL 22 mg/ml protein and 1 µl of reservoir buffer (0.1 M citrate-phosphate buffer, pH 4.2, 40% (v/v) PEG 300). Diffraction data were acquired by an X-ray home source (Rikagu, Sevenoak, UK) and at beamline X12 at the EMBL outstation (Hamburg, Germany).

## Supporting Information

Table S1 **Details of LAMP expression constructs.**
(PDF)

Table S2 **Sequences of the primers listed in Table S1.**
(PDF)
