# Supplementary material for: Streamlining Homogeneous Glycoprotein Production for Biophysical and Structural Applications by Targeted Cell Line Development
Source: PLoS One. 2011 Dec 9;6(12):e27829. doi: 10.1371/journal.pone.0027829 (PMC3235087; doi:10.1371/journal.pone.0027829)
Supplement: Table S1 — Details of LAMP expression constructs. (PDF) [file pone.0027829.s001.pdf]

**Table S1**

Details of LAMP expression constructs

| Protein[a] | Domain[b] | GenBank accession | Construct[c] | PCR primer[d] | Secreted protein detected | Stable cell line |
|---|---|---|---|---|---|---|
| btLAMP2 | lum. | NP_001029742 | nat. SP, 1-368 | 826, 812 | yes | |
| btLAMP2 | lum. | NP_001029742 | Ig SP, 29-368 | 809, 812 | no | |
| btLAMP2 | prox. | NP_001029742 | Ig SP, 217-368 | 811, 812 | no | |
| hLAMP1 | dist. | AAH21288 | Ig SP, 29-195 | 789, 790 | no | |
| hLAMP1 | lum. | AAH21288 | Ig SP, 29-381 | 789, 792 | yes | |
| hLAMP1 | prox. | AAH21288 | Ig SP, 218-381 | 791, 792 | yes | |
| hLAMP1 | dist. | AAH21288 | nat. SP, 1-195 | 821, 790 | no | |
| hLAMP1 | lum. | AAH21288 | nat. SP, 1-381 | 821, 792 | no | |
| hLAMP2 | lum. | AAH02965 | nat. SP, 1-371 | 827, 816 | yes | |
| hLAMP2 | dist. | NP_002285 | Ig SP, 26-193 | 813, 814 | yes | |
| hLAMP2 | dist. | NP_002285 | nat. SP, 1-193 | 827, 814 | yes | |
| hLAMP2 | lum. | AAH02965 | Ig SP, 26-371 | 813, 816 | no | yes |
| hLAMP2 | prox. | AAH02965 | Ig SP, 220-371 | 815, 816 | no | |
| hLAMP3 | dist. | AAH32940 | Ig SP, 28-210 | 817, 818 | no | |
| hLAMP3 | prox. | AAH32940 | Ig SP, 222-381 | 819, 820 | yes | yes |
| hLAMP3 | lum. | AAH32940 | nat. SP, 1-210 | 817, 820 | no | |

| | | | | | | |
|---|---|---|---|---|---|---|
| mLAMP1 | prox. | NP_034814 | Ig SP, 209-370 | 795, 796 | yes | |
| mLAMP1 | dist. | NP_034814 | Ig SP, 25-189 | 793, 794 | yes | |
| mLAMP1 | lum. | NP_034814 | Ig SP, 25-370 | 793, 796 | yes | |
| mLAMP1 | dist. | NP_034814 | nat. SP, 1-189 | 822, 794 | yes | |
| mLAMP1 | lum. | NP_034814 | nat. SP, 1-370 | 822, 796 | yes | |
| mLAMP2 | lum. | NP_001017959 | Ig SP, 26-376 | 801, 804 | no | |
| mLAMP2 | dist. | NP_001017959 | Ig SP, 26-189 | 801, 802 | yes | yes |
| mLAMP2 | prox. | NP_001017959 | Ig SP, 224-376 | 803, 804 | no | |
| mLAMP2 | lum. | NP_001017959 | nat. SP, 1-376 | 824, 804 | yes | yes |
| rLAMP2 | dist. | AAH61990 | Ig SP, 27-189 | 805, 806 | yes | yes |
| rLAMP2 | dist. | AAH61990 | nat. SP, 1-189 | 825, 806 | yes | |
| rLAMP2 | lum. | AAH61990 | Ig SP, 27-372 | 805, 808 | yes | |
| rLAMP2 | prox. | AAH61990 | Ig SP, 220-372 | 807, 808 | no | |
| xlLAMP1 | dist. | AAH77933 | Ig SP, 28-196 | 797, 798 | no | |
| xlLAMP1 | prox. | AAH77933 | Ig SP, 220-381 | 799, 800 | no | |
| xlLAMP1 | dist. | AAH77933 | nat. SP, 1-196 | 823, 798 | yes | |
| xlLAMP1 | lum. | AAH77933 | nat. SP, 1-381 | 797, 800 | yes | |

[a]prefixes: bt=bos taurus, h=human, m=mouse, xl=xenopus laevis, r=rat.

[b]lum.=full luminal domain, prox./dist.=the half proximal/distal to the membrane.

[c]Ig SP=immune globulin signal peptide of SwissProt HVM06_MOUSE, nat. SP=native signal peptide.

[d]PCR primer sequences are listed in Table S2.
