# Supplementary material for: Streamlining Homogeneous Glycoprotein Production for Biophysical and Structural Applications by Targeted Cell Line Development
Source: PLoS One. 2011 Dec 9;6(12):e27829. doi: 10.1371/journal.pone.0027829 (PMC3235087; doi:10.1371/journal.pone.0027829)
Supplement: Table S2 — Sequences of the primers listed in Table S1. (PDF) [file pone.0027829.s002.pdf]

Table S2

Sequences of the primers listed in Table S1.

PCR Primer	Sequence
789	ACCggTgTTCACTCCgCAATgTTTATggTgAAAAATggCA
790	AAAgTACAggTTCTCCCTgTCTTgTTCACAgCgTg
791	ACCggTgTTCACTCCTCTgTggACAAgTACAACgTgA
792	AAAgTACAggTTCTCgCTgTTCTCgTCCAgCAgA
793	ACCggTgTTCACTCCCTCTTTgAggTgAAAAACAATggC
794	AAAgTACAggTTCTCTCCATCCTgTgTgCAgTgT
795	ACCggTgTTCACTCCACTgTATCCAAgTACAATgTTACTggT
796	AAAgTACAggTTCTCgTTgTTACCATCCTgAACACACTC
797	ACCggTgTTCACTCCgTTCAATTTgAAgTgAAAgATggCAA
798	AAAgTACAggTTCTCAATATCCTCACTgCACgTgCT
799	ACCggTgTTCACTCCgTggTgCAgTATTCTgTAAATCgA
800	AAAgTACAggTTCTCCCCgTTTTCATCCAATgAgCA
801	ACCggTgTTCACTCCTTgATAgTTAATTTgACAgATTCAAAgggT
802	AAAgTACAggTTCTCTTggTCTTCTTCACACACTTgTTC
803	ACCggTgTTCACTCCACCgTTggAAACTACAgCATTAg
804	AAAgTACAggTTCTCATCTgCACTgCAgTCTTgAgC
805	ACCggTgTTCACTCCTTgAAACTTAATTTgACAgATTCAAAggg
806	AAAgTACAggTTCTCTTTgTCCTCCTTACACACTTgTTC
821	CCggTCgCCACCATggCggCCCCCggCAgCg
822	CCggTCgCCACCATggCggCCCCCggCgCCC
823	CCggTCgCCACCATgAgTTggAggCAAgTCAAgATgC
824	CCggTCgCCACCATgTgCCTCTCTCCggTTAAAgg
825	CCggTCgCCACCATgCgCCTCCTCTCTCCgg
826	CCggTCgCCACCATggTgTgCTTCCgCCTCg
827	CCggTCgCCACCATggTgTgCTTCCgCCTCTTC
807	ACCggTgTTCACTCCACggTTggAAACTACACCATTTC
808	AAAgTACAggTTCTCATCTgCACTgCAgTCTTgAgC

809	ACCggTgTTCACTCCTTggAACTTAATTTgACAgATTCATCAAAC
811	ACCggTgTTCACTCCgTCggATCCTATTCAgTTgTAAATAgC
812	AAAgTACAggTTCTCATCCAgCgAACACTCTTggg
813	ACCggTgTTCACTCCTCTTATgCATTggAACTTAATTTgACAgA
814	AAAgTACAggTTCTCTTTgTCTTTATCACACAggAACTCATT
815	ACCggTgTTCACTCCgCTggAACCTATTCAgTTAATAATggC
816	AAAgTACAggTTCTCATCTgCACTgCAgTCTTgAgC
817	ACCggTgTTCACTCCAAAgCATTTCCAggAACCAgAg
818	AAAgTACAggTTCTCCgTggAggCAggTgCA
819	ACCggTgTTCACTCCTCAgTCAAgACTggAATTTATCAggT
820	AAAgTACAggTTCTCTgTgTAgTCAgACgAgCACTC
